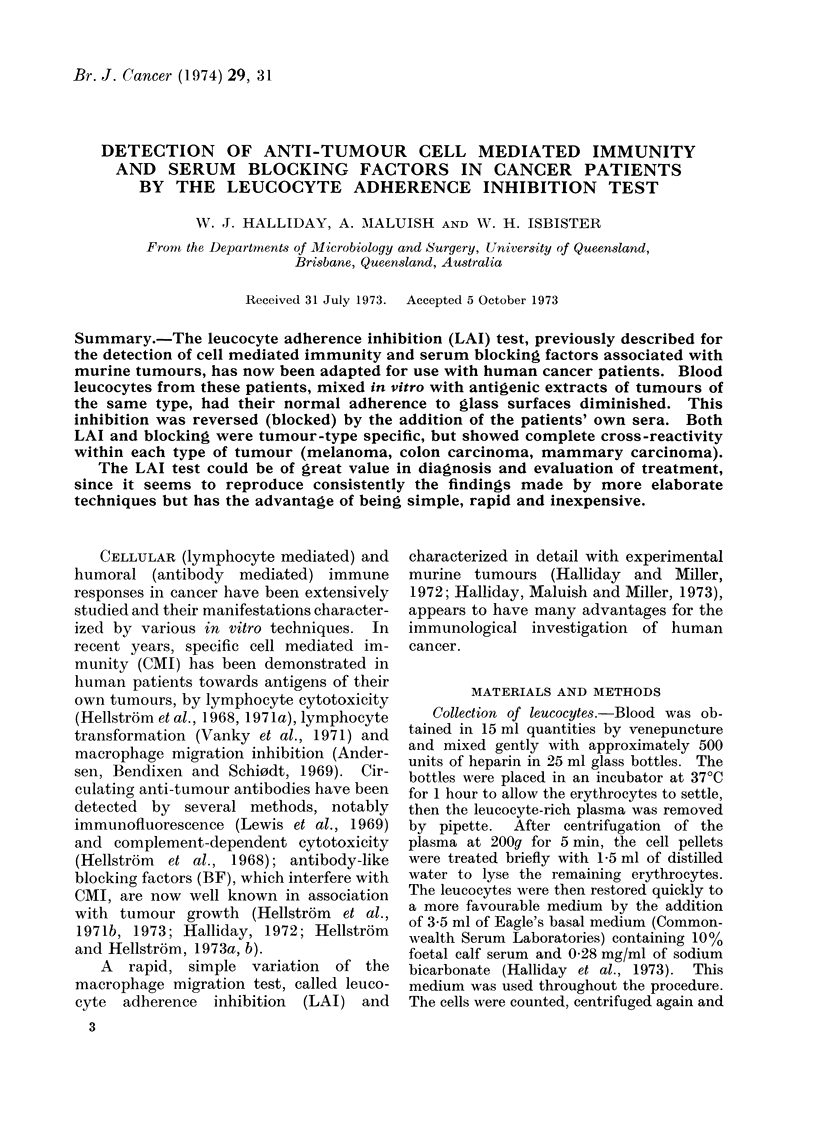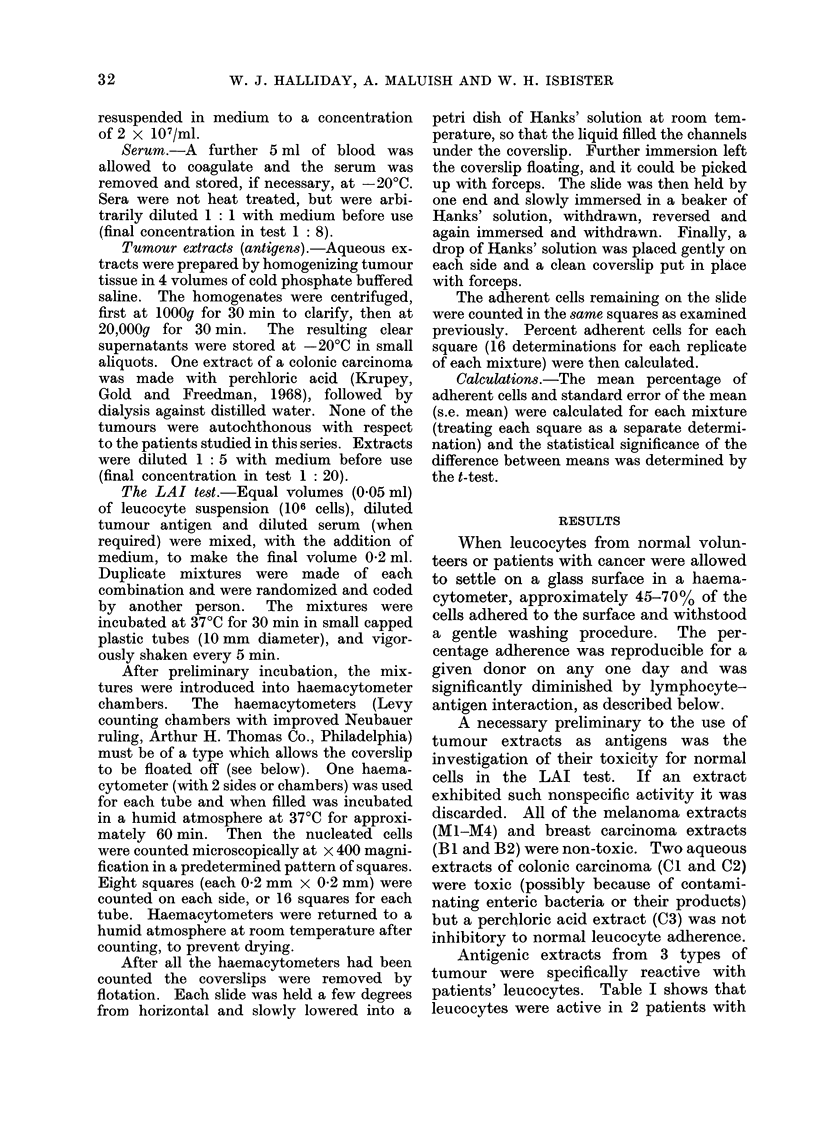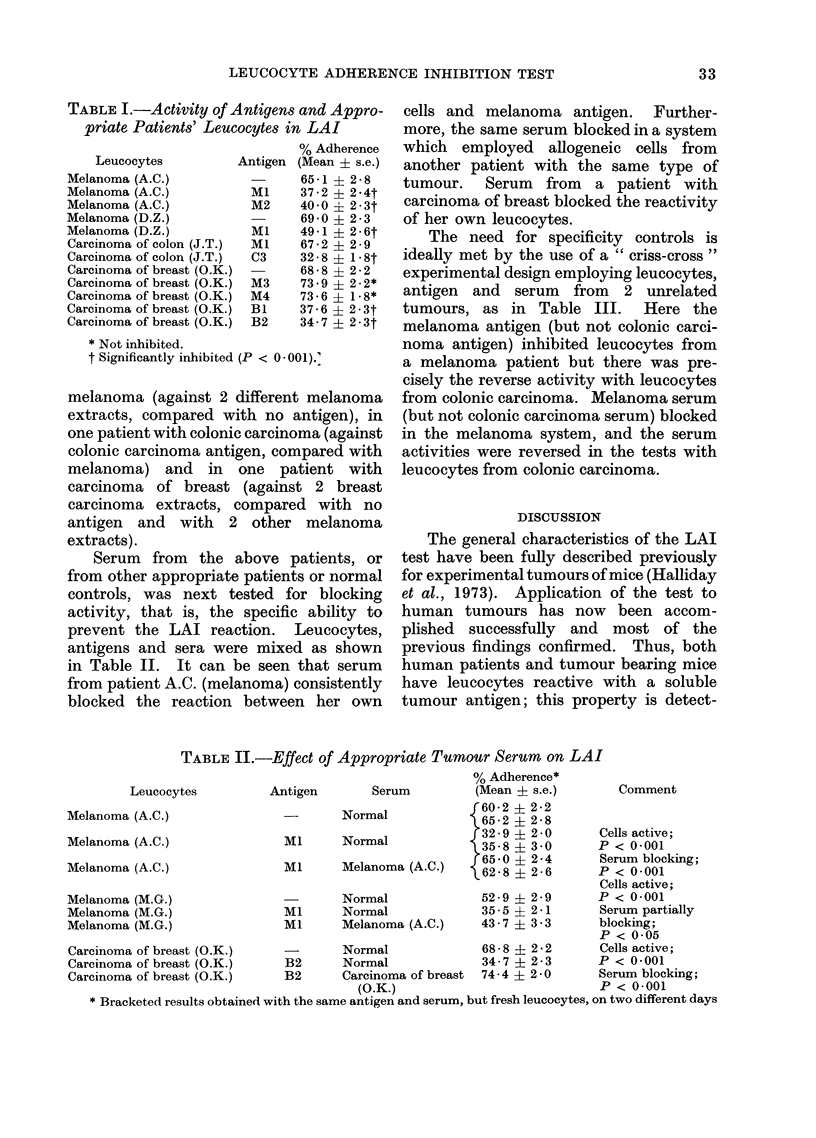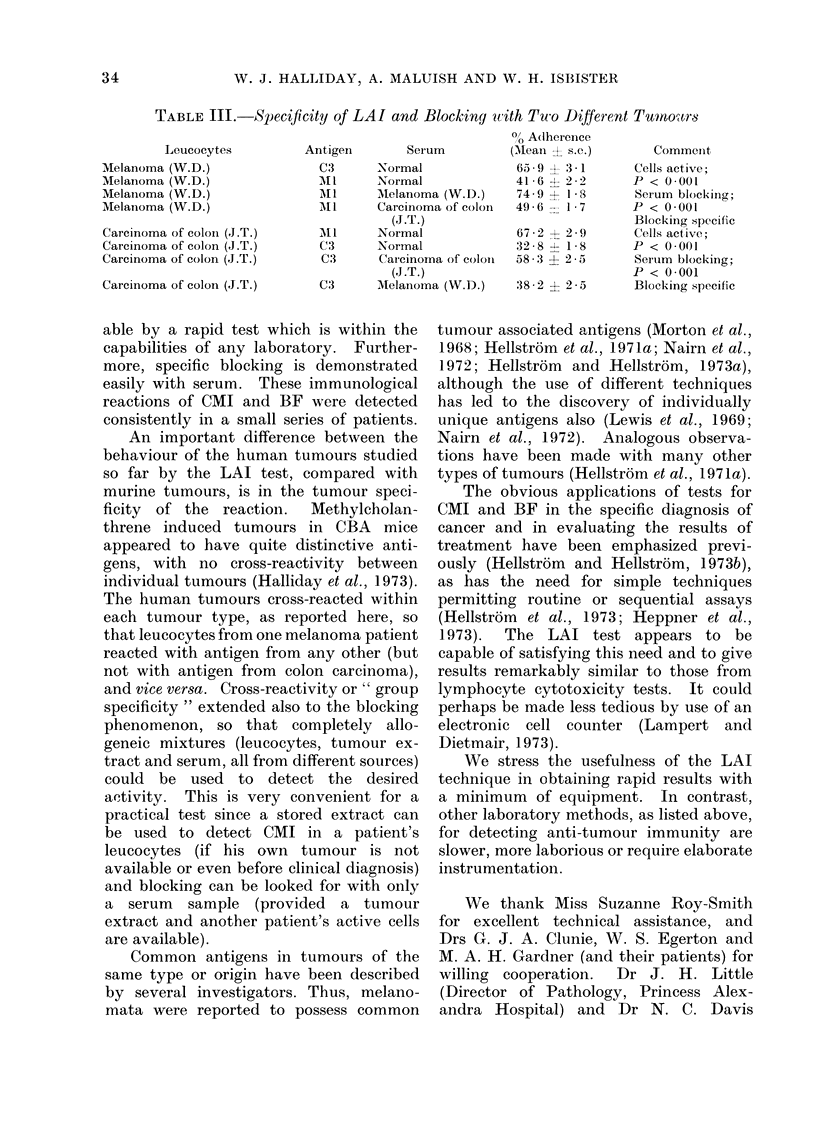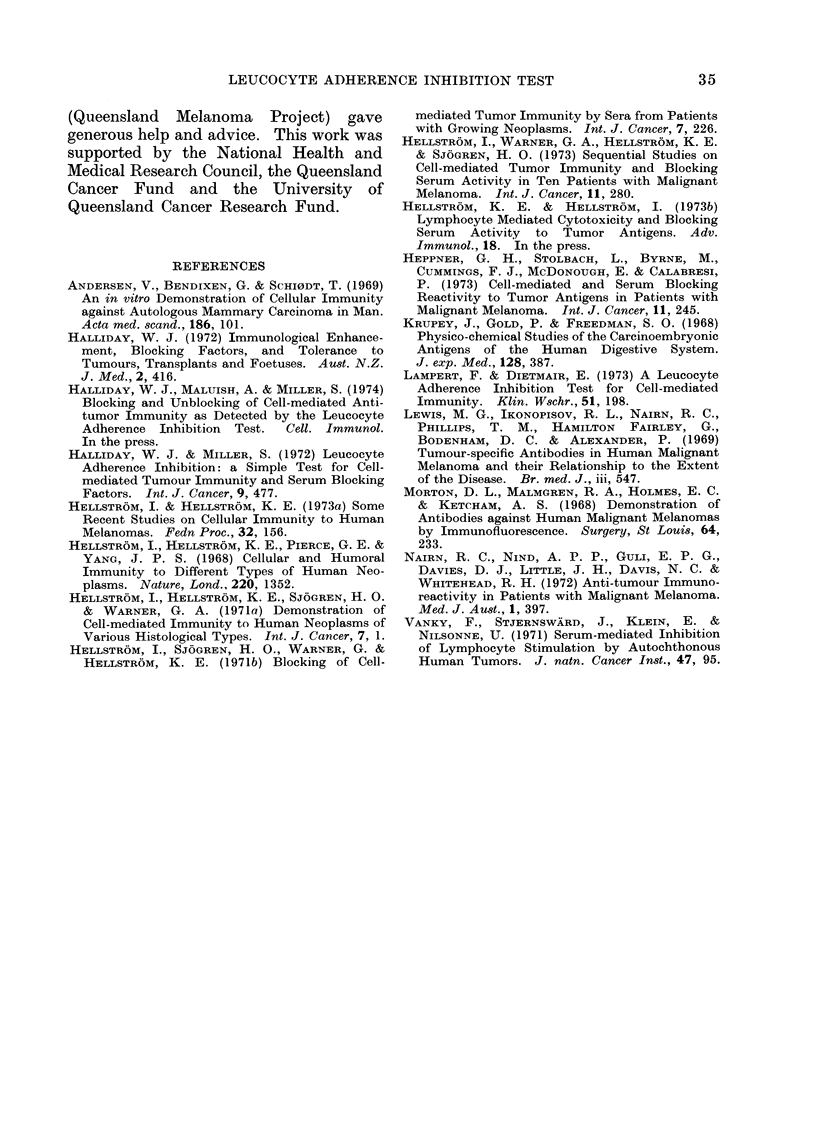# Detection of Anti-tumour Cell Mediated Immunity and Serum Blocking Factors in Cancer Patients by the Leucocyte Adherence Inhibition Test

**DOI:** 10.1038/bjc.1974.4

**Published:** 1974-01

**Authors:** W. J. Halliday, A. Maluish, W. H. Isbister

## Abstract

The leucocyte adherence inhibition (LAI) test, previously described for the detection of cell mediated immunity and serum blocking factors associated with murine tumours, has now been adapted for use with human cancer patients. Blood leucocytes from these patients, mixed *in vitro* with antigenic extracts of tumours of the same type, had their normal adherence to glass surfaces diminished. This inhibition was reversed (blocked) by the addition of the patients' own sera. Both LAI and blocking were tumour-type specific, but showed complete cross-reactivity within each type of tumour (melanoma, colon carcinoma, mammary carcinoma).

The LAI test could be of great value in diagnosis and evaluation of treatment, since it seems to reproduce consistently the findings made by more elaborate techniques but has the advantage of being simple, rapid and inexpensive.


*Br. J. Cancer* (1974) **29**, 31

### DETECTION OF ANTI-TUMOUR CELL MEDIATED IMMUNITY AND SERUM BLOCKING FACTORS IN CANCER PATIENTS BY THE LEUCOCYTE ADHERENCE INHIBITION TEST

W. J. HALLIDAY, A. MALUISH AND W. H. ISBISTER

*From the Departments of Microbiology and Surgery, University of Queensland, Brisbane, Queensland, Australia*

Received 31 July 1973. Accepted 5 October 1973

**Summary.—The leucocyte adherence inhibition (LAI) test, previously described for the detection of cell mediated immunity and serum blocking factors associated with murine tumours, has now been adapted for use with human cancer patients. Blood leucocytes from these patients, mixed *in vitro* with antigenic extracts of tumours of the same type, had their normal adherence to glass surfaces diminished. This inhibition was reversed (blocked) by the addition of the patients' own sera. Both LAI and blocking were tumour-type specific, but showed complete cross-reactivity within each type of tumour (melanoma, colon carcinoma, mammary carcinoma).**

**The LAI test could be of great value in diagnosis and evaluation of treatment, since it seems to reproduce consistently the findings made by more elaborate techniques but has the advantage of being simple, rapid and inexpensive.**

CELLULAR (lymphocyte mediated) and humoral (antibody mediated) immune responses in cancer have been extensively studied and their manifestations characterized by various *in vitro* techniques. In recent years, specific cell mediated immunity (CMI) has been demonstrated in human patients towards antigens of their own tumours, by lymphocyte cytotoxicity (Hellström *et al.*, 1968, 1971*a*), lymphocyte transformation (Vanky *et al.*, 1971) and macrophage migration inhibition (Andersen, Bendixen and Schiødt, 1969). Circulating anti-tumour antibodies have been detected by several methods, notably immunofluorescence (Lewis *et al.*, 1969) and complement-dependent cytotoxicity (Hellström *et al.*, 1968); antibody-like blocking factors (BF), which interfere with CMI, are now well known in association with tumour growth (Hellström *et al.*, 1971*b*, 1973; Halliday, 1972; Hellström and Hellström, 1973*a*, *b*).

A rapid, simple variation of the macrophage migration test, called leucocyte adherence inhibition (LAI) and characterized in detail with experimental murine tumours (Halliday and Miller, 1972; Halliday, Maluish and Miller, 1973), appears to have many advantages for the immunological investigation of human cancer.

### MATERIALS AND METHODS

*Collection of leucocytes.*—Blood was obtained in 15 ml quantities by venepuncture and mixed gently with approximately 500 units of heparin in 25 ml glass bottles. The bottles were placed in an incubator at 37°C for 1 hour to allow the erythrocytes to settle, then the leucocyte-rich plasma was removed by pipette. After centrifugation of the plasma at 200*g* for 5 min, the cell pellets were treated briefly with 1·5 ml of distilled water to lyse the remaining erythrocytes. The leucocytes were then restored quickly to a more favourable medium by the addition of 3·5 ml of Eagle's basal medium (Commonwealth Serum Laboratories) containing 10% foetal calf serum and 0·28 mg/ml of sodium bicarbonate (Halliday *et al.*, 1973). This medium was used throughout the procedure. The cells were counted, centrifuged again and resuspended in medium to a concentration of $2 \times 10^7$/ml.

*Serum.*—A further 5 ml of blood was allowed to coagulate and the serum was removed and stored, if necessary, at −20°C. Sera were not heat treated, but were arbitrarily diluted 1 : 1 with medium before use (final concentration in test 1 : 8).

*Tumour extracts (antigens).*—Aqueous extracts were prepared by homogenizing tumour tissue in 4 volumes of cold phosphate buffered saline. The homogenates were centrifuged, first at 1000*g* for 30 min to clarify, then at 20,000*g* for 30 min. The resulting clear supernatants were stored at −20°C in small aliquots. One extract of a colonic carcinoma was made with perchloric acid (Krupey, Gold and Freedman, 1968), followed by dialysis against distilled water. None of the tumours were autochthonous with respect to the patients studied in this series. Extracts were diluted 1 : 5 with medium before use (final concentration in test 1 : 20).

*The LAI test.*—Equal volumes (0·05 ml) of leucocyte suspension ($10^6$ cells), diluted tumour antigen and diluted serum (when required) were mixed, with the addition of medium, to make the final volume 0·2 ml. Duplicate mixtures were made of each combination and were randomized and coded by another person. The mixtures were incubated at 37°C for 30 min in small capped plastic tubes (10 mm diameter), and vigorously shaken every 5 min.

After preliminary incubation, the mixtures were introduced into haemacytometer chambers. The haemacytometers (Levy counting chambers with improved Neubauer ruling, Arthur H. Thomas Co., Philadelphia) must be of a type which allows the coverslip to be floated off (see below). One haemacytometer (with 2 sides or chambers) was used for each tube and when filled was incubated in a humid atmosphere at 37°C for approximately 60 min. Then the nucleated cells were counted microscopically at ×400 magnification in a predetermined pattern of squares. Eight squares (each 0·2 mm × 0·2 mm) were counted on each side, or 16 squares for each tube. Haemacytometers were returned to a humid atmosphere at room temperature after counting, to prevent drying.

After all the haemacytometers had been counted the coverslips were removed by flotation. Each slide was held a few degrees from horizontal and slowly lowered into a petri dish of Hanks' solution at room temperature, so that the liquid filled the channels under the coverslip. Further immersion left the coverslip floating, and it could be picked up with forceps. The slide was then held by one end and slowly immersed in a beaker of Hanks' solution, withdrawn, reversed and again immersed and withdrawn. Finally, a drop of Hanks' solution was placed gently on each side and a clean coverslip put in place with forceps.

The adherent cells remaining on the slide were counted in the *same* squares as examined previously. Percent adherent cells for each square (16 determinations for each replicate of each mixture) were then calculated.

*Calculations.*—The mean percentage of adherent cells and standard error of the mean (s.e. mean) were calculated for each mixture (treating each square as a separate determination) and the statistical significance of the difference between means was determined by the *t*-test.

### RESULTS

When leucocytes from normal volunteers or patients with cancer were allowed to settle on a glass surface in a haemacytometer, approximately 45–70% of the cells adhered to the surface and withstood a gentle washing procedure. The percentage adherence was reproducible for a given donor on any one day and was significantly diminished by lymphocyte–antigen interaction, as described below.

A necessary preliminary to the use of tumour extracts as antigens was the investigation of their toxicity for normal cells in the LAI test. If an extract exhibited such nonspecific activity it was discarded. All of the melanoma extracts (M1–M4) and breast carcinoma extracts (B1 and B2) were non-toxic. Two aqueous extracts of colonic carcinoma (C1 and C2) were toxic (possibly because of contaminating enteric bacteria or their products) but a perchloric acid extract (C3) was not inhibitory to normal leucocyte adherence.

Antigenic extracts from 3 types of tumour were specifically reactive with patients' leucocytes. Table I shows that leucocytes were active in 2 patients with

TABLE I.—*Activity of Antigens and Appropriate Patients' Leucocytes in LAI*

| Leucocytes | Antigen | % Adherence (Mean ± s.e.) |
|---|---|---|
| Melanoma (A.C.) | — | 65·1 ± 2·8 |
| Melanoma (A.C.) | M1 | 37·2 ± 2·4† |
| Melanoma (A.C.) | M2 | 40·0 ± 2·3† |
| Melanoma (D.Z.) | — | 69·0 ± 2·3 |
| Melanoma (D.Z.) | M1 | 49·1 ± 2·6† |
| Carcinoma of colon (J.T.) | M1 | 67·2 ± 2·9 |
| Carcinoma of colon (J.T.) | C3 | 32·8 ± 1·8† |
| Carcinoma of breast (O.K.) | — | 68·8 ± 2·2 |
| Carcinoma of breast (O.K.) | M3 | 73·9 ± 2·2* |
| Carcinoma of breast (O.K.) | M4 | 73·6 ± 1·8* |
| Carcinoma of breast (O.K.) | B1 | 37·6 ± 2·3† |
| Carcinoma of breast (O.K.) | B2 | 34·7 ± 2·3† |

* Not inhibited.
† Significantly inhibited ($P < 0{\cdot}001$).

melanoma (against 2 different melanoma extracts, compared with no antigen), in one patient with colonic carcinoma (against colonic carcinoma antigen, compared with melanoma) and in one patient with carcinoma of breast (against 2 breast carcinoma extracts, compared with no antigen and with 2 other melanoma extracts).

Serum from the above patients, or from other appropriate patients or normal controls, was next tested for blocking activity, that is, the specific ability to prevent the LAI reaction. Leucocytes, antigens and sera were mixed as shown in Table II. It can be seen that serum from patient A.C. (melanoma) consistently blocked the reaction between her own cells and melanoma antigen. Furthermore, the same serum blocked in a system which employed allogeneic cells from another patient with the same type of tumour. Serum from a patient with carcinoma of breast blocked the reactivity of her own leucocytes.

The need for specificity controls is ideally met by the use of a " criss-cross " experimental design employing leucocytes, antigen and serum from 2 unrelated tumours, as in Table III. Here the melanoma antigen (but not colonic carcinoma antigen) inhibited leucocytes from a melanoma patient but there was precisely the reverse activity with leucocytes from colonic carcinoma. Melanoma serum (but not colonic carcinoma serum) blocked in the melanoma system, and the serum activities were reversed in the tests with leucocytes from colonic carcinoma.

### DISCUSSION

The general characteristics of the LAI test have been fully described previously for experimental tumours of mice (Halliday *et al.*, 1973). Application of the test to human tumours has now been accomplished successfully and most of the previous findings confirmed. Thus, both human patients and tumour bearing mice have leucocytes reactive with a soluble tumour antigen; this property is detect-

TABLE II.—*Effect of Appropriate Tumour Serum on LAI*

| Leucocytes | Antigen | Serum | % Adherence* (Mean ± s.e.) | Comment |
|---|---|---|---|---|
| Melanoma (A.C.) | — | Normal | {60·2 ± 2·2<br>65·2 ± 2·8 | |
| Melanoma (A.C.) | M1 | Normal | {32·9 ± 2·0<br>35·8 ± 3·0 | Cells active; $P < 0{\cdot}001$ |
| Melanoma (A.C.) | M1 | Melanoma (A.C.) | {65·0 ± 2·4<br>62·8 ± 2·6 | Serum blocking; $P < 0{\cdot}001$ |
| Melanoma (M.G.) | — | Normal | 52·9 ± 2·9 | Cells active; $P < 0{\cdot}001$ |
| Melanoma (M.G.) | M1 | Normal | 35·5 ± 2·1 | Serum partially |
| Melanoma (M.G.) | M1 | Melanoma (A.C.) | 43·7 ± 3·3 | blocking; $P < 0{\cdot}05$ |
| Carcinoma of breast (O.K.) | — | Normal | 68·8 ± 2·2 | Cells active; |
| Carcinoma of breast (O.K.) | B2 | Normal | 34·7 ± 2·3 | $P < 0{\cdot}001$ |
| Carcinoma of breast (O.K.) | B2 | Carcinoma of breast (O.K.) | 74·4 ± 2·0 | Serum blocking; $P < 0{\cdot}001$ |

* Bracketed results obtained with the same antigen and serum, but fresh leucocytes, on two different days

TABLE III.—*Specificity of LAI and Blocking with Two Different Tumours*

| Leucocytes | Antigen | Serum | % Adherence (Mean ± s.e.) | Comment |
|---|---|---|---|---|
| Melanoma (W.D.) | C3 | Normal | 65·9 ± 3·1 | Cells active; |
| Melanoma (W.D.) | M1 | Normal | 41·6 ± 2·2 | $P < 0{\cdot}001$ |
| Melanoma (W.D.) | M1 | Melanoma (W.D.) | 74·9 ± 1·8 | Serum blocking; |
| Melanoma (W.D.) | M1 | Carcinoma of colon (J.T.) | 49·6 ± 1·7 | $P < 0{\cdot}001$ Blocking specific |
| Carcinoma of colon (J.T.) | M1 | Normal | 67·2 ± 2·9 | Cells active; |
| Carcinoma of colon (J.T.) | C3 | Normal | 32·8 ± 1·8 | $P < 0{\cdot}001$ |
| Carcinoma of colon (J.T.) | C3 | Carcinoma of colon (J.T.) | 58·3 ± 2·5 | Serum blocking; $P < 0{\cdot}001$ |
| Carcinoma of colon (J.T.) | C3 | Melanoma (W.D.) | 38·2 ± 2·5 | Blocking specific |

able by a rapid test which is within the capabilities of any laboratory. Furthermore, specific blocking is demonstrated easily with serum. These immunological reactions of CMI and BF were detected consistently in a small series of patients.

An important difference between the behaviour of the human tumours studied so far by the LAI test, compared with murine tumours, is in the tumour specificity of the reaction. Methylcholanthrene induced tumours in CBA mice appeared to have quite distinctive antigens, with no cross-reactivity between individual tumours (Halliday *et al.*, 1973). The human tumours cross-reacted within each tumour type, as reported here, so that leucocytes from one melanoma patient reacted with antigen from any other (but not with antigen from colon carcinoma), and *vice versa*. Cross-reactivity or " group specificity " extended also to the blocking phenomenon, so that completely allogeneic mixtures (leucocytes, tumour extract and serum, all from different sources) could be used to detect the desired activity. This is very convenient for a practical test since a stored extract can be used to detect CMI in a patient's leucocytes (if his own tumour is not available or even before clinical diagnosis) and blocking can be looked for with only a serum sample (provided a tumour extract and another patient's active cells are available).

Common antigens in tumours of the same type or origin have been described by several investigators. Thus, melanomata were reported to possess common tumour associated antigens (Morton *et al.*, 1968; Hellström *et al.*, 1971*a*; Nairn *et al.*, 1972; Hellström and Hellström, 1973*a*), although the use of different techniques has led to the discovery of individually unique antigens also (Lewis *et al.*, 1969; Nairn *et al.*, 1972). Analogous observations have been made with many other types of tumours (Hellström *et al.*, 1971*a*).

The obvious applications of tests for CMI and BF in the specific diagnosis of cancer and in evaluating the results of treatment have been emphasized previously (Hellström and Hellström, 1973*b*), as has the need for simple techniques permitting routine or sequential assays (Hellström *et al.*, 1973; Heppner *et al.*, 1973). The LAI test appears to be capable of satisfying this need and to give results remarkably similar to those from lymphocyte cytotoxicity tests. It could perhaps be made less tedious by use of an electronic cell counter (Lampert and Dietmair, 1973).

We stress the usefulness of the LAI technique in obtaining rapid results with a minimum of equipment. In contrast, other laboratory methods, as listed above, for detecting anti-tumour immunity are slower, more laborious or require elaborate instrumentation.

We thank Miss Suzanne Roy-Smith for excellent technical assistance, and Drs G. J. A. Clunie, W. S. Egerton and M. A. H. Gardner (and their patients) for willing cooperation. Dr J. H. Little (Director of Pathology, Princess Alexandra Hospital) and Dr N. C. Davis

(Queensland Melanoma Project) gave generous help and advice. This work was supported by the National Health and Medical Research Council, the Queensland Cancer Fund and the University of Queensland Cancer Research Fund.

### REFERENCES

ANDERSEN, V., BENDIXEN, G. & SCHIØDT, T. (1969) An *in vitro* Demonstration of Cellular Immunity against Autologous Mammary Carcinoma in Man. *Acta med. scand.*, **186**, 101.

HALLIDAY, W. J. (1972) Immunological Enhancement, Blocking Factors, and Tolerance to Tumours, Transplants and Foetuses. *Aust. N.Z. J. Med.*, **2**, 416.

HALLIDAY, W. J., MALUISH, A. & MILLER, S. (1974) Blocking and Unblocking of Cell-mediated Antitumor Immunity as Detected by the Leucocyte Adherence Inhibition Test. *Cell. Immunol.* In the press.

HALLIDAY, W. J. & MILLER, S. (1972) Leucocyte Adherence Inhibition: a Simple Test for Cell-mediated Tumour Immunity and Serum Blocking Factors. *Int. J. Cancer*, **9**, 477.

HELLSTRÖM, I. & HELLSTRÖM, K. E. (1973*a*) Some Recent Studies on Cellular Immunity to Human Melanomas. *Fedn Proc.*, **32**, 156.

HELLSTRÖM, I., HELLSTRÖM, K. E., PIERCE, G. E. & YANG, J. P. S. (1968) Cellular and Humoral Immunity to Different Types of Human Neoplasms. *Nature, Lond.*, **220**, 1352.

HELLSTRÖM, I., HELLSTRÖM, K. E., SJÖGREN, H. O. & WARNER, G. A. (1971*a*) Demonstration of Cell-mediated Immunity to Human Neoplasms of Various Histological Types. *Int. J. Cancer*, **7**, 1.

HELLSTRÖM, I., SJÖGREN, H. O., WARNER, G. & HELLSTRÖM, K. E. (1971*b*) Blocking of Cell-mediated Tumor Immunity by Sera from Patients with Growing Neoplasms. *Int. J. Cancer*, **7**, 226.

HELLSTRÖM, I., WARNER, G. A., HELLSTRÖM, K. E. & SJÖGREN, H. O. (1973) Sequential Studies on Cell-mediated Tumor Immunity and Blocking Serum Activity in Ten Patients with Malignant Melanoma. *Int. J. Cancer*, **11**, 280.

HELLSTRÖM, K. E. & HELLSTRÖM, I. (1973*b*) Lymphocyte Mediated Cytotoxicity and Blocking Serum Activity to Tumor Antigens. *Adv. Immunol.*, **18**. In the press.

HEPPNER, G. H., STOLBACH, L., BYRNE, M., CUMMINGS, F. J., McDONOUGH, E. & CALABRESI, P. (1973) Cell-mediated and Serum Blocking Reactivity to Tumor Antigens in Patients with Malignant Melanoma. *Int. J. Cancer*, **11**, 245.

KRUPEY, J., GOLD, P. & FREEDMAN, S. O. (1968) Physico-chemical Studies of the Carcinoembryonic Antigens of the Human Digestive System. *J. exp. Med.*, **128**, 387.

LAMPERT, F. & DIETMAIR, E. (1973) A Leucocyte Adherence Inhibition Test for Cell-mediated Immunity. *Klin. Wschr.*, **51**, 198.

LEWIS, M. G., IKONOPISOV, R. L., NAIRN, R. C., PHILLIPS, T. M., HAMILTON FAIRLEY, G., BODENHAM, D. C. & ALEXANDER, P. (1969) Tumour-specific Antibodies in Human Malignant Melanoma and their Relationship to the Extent of the Disease. *Br. med. J.*, iii, 547.

MORTON, D. L., MALMGREN, R. A., HOLMES, E. C. & KETCHAM, A. S. (1968) Demonstration of Antibodies against Human Malignant Melanomas by Immunofluorescence. *Surgery, St Louis*, **64**, 233.

NAIRN, R. C., NIND, A. P. P., GULI, E. P. G., DAVIES, D. J., LITTLE, J. H., DAVIS, N. C. & WHITEHEAD, R. H. (1972) Anti-tumour Immunoreactivity in Patients with Malignant Melanoma. *Med. J. Aust.*, **1**, 397.

VANKY, F., STJERNSWÄRD, J., KLEIN, E. & NILSONNE, U. (1971) Serum-mediated Inhibition of Lymphocyte Stimulation by Autochthonous Human Tumors. *J. natn. Cancer Inst.*, **47**, 95.